# Ketamine and Electroconvulsive Therapy: Better Together?

**DOI:** 10.1192/j.eurpsy.2022.1445

**Published:** 2022-09-01

**Authors:** A. Fraga, B. Mesquita, J. Facucho-Oliveira, P. Espada-Santos, M. Albuquerque, R. Neves, A. Moutinho

**Affiliations:** 1 Hospital de Cascais, Psychiatry, Alcabideche, Portugal; 2 Hospital de Monsanto, Psychiatry, Lisboa, Portugal

**Keywords:** Ketamine, Depression, Treatment-resistant depression, Electoconvulsive therapy

## Abstract

**Introduction:**

Major depressive disorder (MDD) is a highly prevalent clinical condition with a leading cause of disability worldwide. The currently available therapeutic agents have important limitations regarding side effects, partial or non-responsiveness. Patients are considered to have treatment-resistant depression (TRD) if there is no effect or minimal effectiveness after receiving adequate dose-duration use of antidepressants from two different categories. For this patients, electroconvulsive therapy (ECT) can be a treatment option and new therapies appear to tackle TRD like ketamine, a dissociative anesthetic and analgesic.

**Objectives:**

The authors elaborate a narrative literature review to understand if ketamine might enhance the antidepressant efficacy of ECT.

**Methods:**

PubMed database searched using the terms “Electroconvulsive therapy”, “ketamine” and “treatment-resistant depression”.

**Results:**

ECT is currently recommended as an end-line therapy for TRD. Memory impairment after ECT could be a consequence of indiscriminate activation or saturation of glutamate receptors during the treatment, disrupting hippocampal plasticity involved in memory. Ketamine inhibits N-methyl-d-aspartate (NMDA) receptors, while stimulating glutamate release and was proposed as an ECT adjuvant, might reduce cognitive adverse effects, time until response/ remission and inclusively improve response rates to ECT.

However, response and remission rates of ketamine in ECT showed no significant difference from the comparator groups and was associated with higher rates of psychiatric and cardiovascular adverse events.

**Conclusions:**

The results did not support the use the combination of ketamine and propofol as anesthetic agents for ECT in patients with MDD. However, further studies are needed to investigate the beneficial clinical and cognitive effects of ketamine alone in ECT settings.

**Disclosure:**

No significant relationships.

